# A Rare Case of Intramedullary Juvenile Xanthogranuloma of the Cervical Spine in an Adult: Cytological Features Aiding Diagnosis

**DOI:** 10.7759/cureus.82068

**Published:** 2025-04-11

**Authors:** Dung A Vu, Motona Kumagai, Yao Liu, Akihiro Shioya, Sohsuke Yamada

**Affiliations:** 1 Department of Pathology and Laboratory Medicine, Kanazawa Medical University, Uchinada, JPN; 2 Department of Joint Surgery, Vietnam Military Medical University, Hanoi, VNM; 3 Department of Pathology, Kanazawa Medical University Hospital, Uchinada, JPN; 4 Department of Pathology, The Fourth Hospital of Hebei Medical University, Shijiazhuang, CHN

**Keywords:** cervical spine, cytopathology, diagnosis, juvenile xanthogranuloma, non-langerhans cell

## Abstract

Juvenile xanthogranuloma (JXG), the most common non-Langerhans cell histiocytosis (non-LCH), typically manifests as a benign cutaneous condition in children but is rare in adults, with extracutaneous cervical spine involvement being uncommon. To the best of our knowledge, we present the first case of intramedullary cervical spinal JXG in a 39-year-old man with persistent right upper extremity numbness. MRI identified a lesion at C3-C4, and surgical resection revealed a 7-mm yellow-whitish nodule. Histopathology demonstrated foamy histiocytes, multinucleated giant cells, and inflammatory infiltrates, with immunohistochemistry positive for CD68, CD163, and vimentin, and negative for CD1a and S-100 protein, distinguishing it from LCH. Cytopathology was instrumental, revealing low cellularity, mononuclear histiocytes, and CD68-positive granules, offering definitive diagnostic precision in this rare presentation. This case underscores cytopathology’s critical role as an essential tool in accurately diagnosing atypical JXG manifestations.

## Introduction

Juvenile xanthogranuloma (JXG), the most prevalent type of non-Langerhans cell histiocytosis (non-LCH), primarily affects the skin in children. It is considered a benign condition, and almost all patients experience spontaneous regression [1]. In contrast, JXG is infrequent in adults, with its incidence peaking among individuals in their late 20s to early 30s [1,2]. These lesions are extracutaneous in location; in particular, lesions of the cervical spine are extremely rare. Only five previous cases of JXG involving the cervical spine with JXG [1-5] and one case showed a spinal intramedullary lesion in the lumbar spine [6]. The typical histological characteristics of JXG include abundant foamy cells, non-foamy mononuclear or multinucleated giant cells, and multiple cholesterol crystals in the lesion [7]. However, the presence of Touton giant cells has not always been observed. The diagnosis usually relies on histopathology, and cystic findings in JXG have been described in a very small number of cases [8]. Due to the rarity of the lesion, its cytological features have not been clearly described. In 1996, Grenko et al. were the first to describe the cytological characteristics of deep JXG in two pediatric patients [8]. Owing to insufficient awareness of this condition among cytopathologists, it may be overlooked during examinations. To the best of our knowledge, we herein present the first report of intramedullary spinal JXG of the cervical cord in a 39-year-old man, together with the detailed diagnostic cytologic features. A comprehensive understanding of the distinctive cytology of JXG is essential for an accurate diagnosis.

## Case presentation

**Figure 1:**
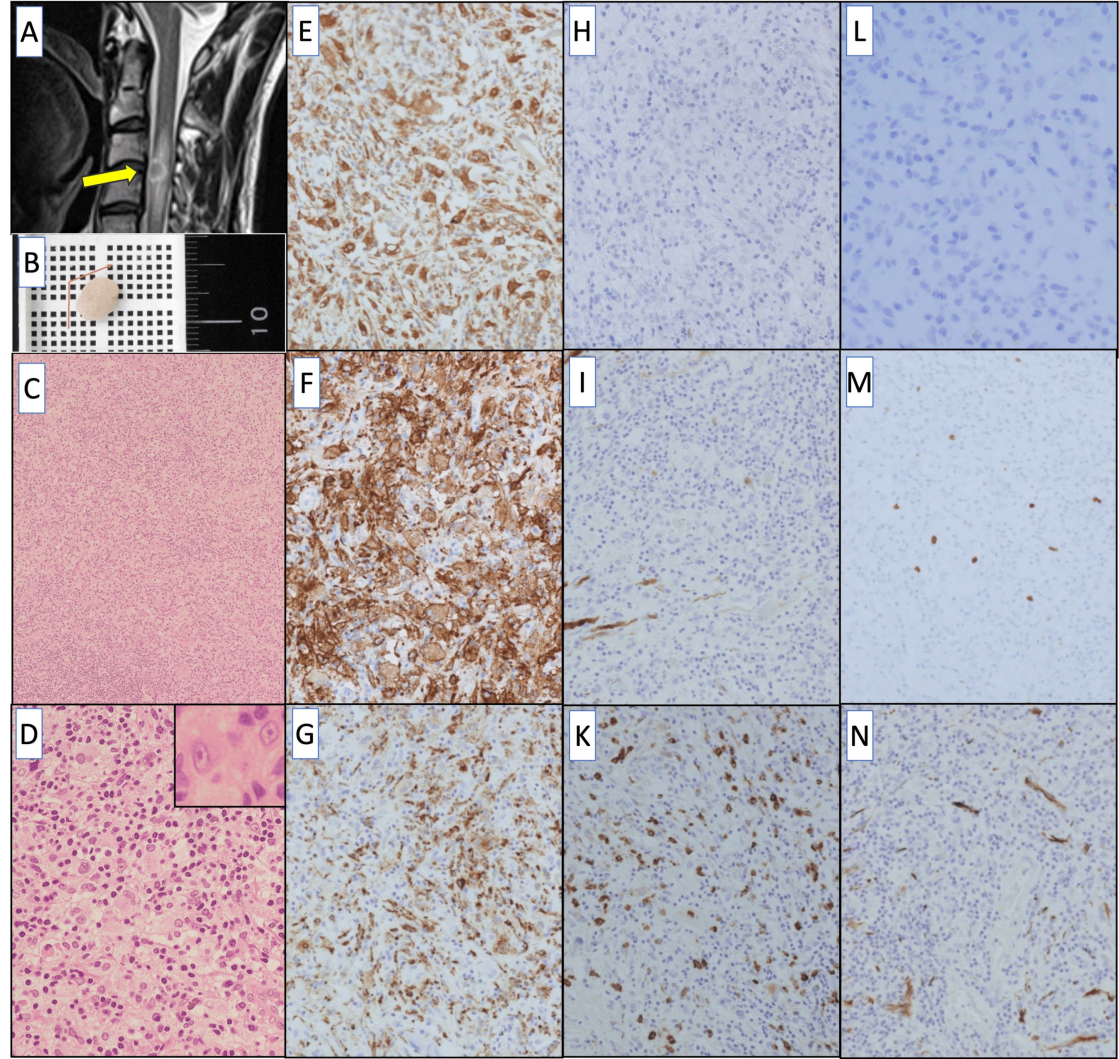
Imaging and histopathology findings. (A) Sagittal MRI T2-weighted image shows isointense mass (yellow arrow) in the spinal canal at the C3-C4; a midline-to-left oval-shaped tumor is observed on the ventral side within the dura mater. The area surrounding the mass exhibits high-tensity signals. (B) Macroscopically, the tumor measures slightly less than 1 cm. (C) Alongside histiocyte-like cells, chronic inflammatory cell infiltration, including lymphocytes, is also observed (H&E staining; ×10). (D) The histiocyte-like cells exhibit foamy to eosinophilic, abundant cytoplasm and proliferate, with round to oval nuclei showing minimal atypia (H&E staining; ×40, Inset; x200). (E, F, G) The tumor cells display significant immuno-positivity for vimentin (E), CD163 (F), and CD68 (G) (Immunohistochemical staining; ×20). (H, I, K) The tumor cells showed immuno-negativity for CD1a (H), S-100 protein (I), and lysozyme (K) (Immunohistochemical staining; ×20). (L) The tumor cells were immunohistochemically negative for Langerin (Immunohistochemical staining; ×40). (M) The Ki67 labeling index reaches much less than 5% (Immunohistochemical staining; ×20). (N) The tumor cells are immuno-negative for glial fibrillary acidic protein (GFAP) (Immunohistochemical staining; ×20).

A 39-year-old man was hospitalized with the chief complaint of unexplained numbness in the right upper extremity that persisted for four months. A sagittal spinal MRI scan revealed a lesion at the C3-C4 region of the cervical spinal cord, either compressing from the anterior side or located within the spinal cord (Figure 1A). A separate positron emission tomography-computed tomography (PET-CT) scan did not detect any other lesions. Clinically, a benign extramedullary tumor was suspected, although the diagnosis was challenging. Tumor resection revealed a 7-mm, oval, yellow-whitish nodule within the spinal cord (Figure 1B), with a smooth surface and 2-mm fragments.

Histologically, small lymphocytes, hyalinized small blood vessels, and fibrous tissues were observed in the background (Figure 1C). Additionally, chronic infiltration of inflammatory cells (e.g., lymphocytes) was observed, along with histiocyte-like cells (Figure 1D). Histiocyte-like cells with foamy to eosinophilic and broad cytoplasm proliferated, ranging from mononuclear to multinucleated forms. The nuclei were round to oval in shape, uniform in size, and sometimes indented or resembled coffee beans. No evidence of mast cells or histiocytic cells exhibiting grooved nuclei characteristic of Langerhans cells was observed. The cytoplasm had ill-defined boundaries. However, mitotic activity was exceedingly rare.

Immunohistochemical staining revealed a low Ki-67 labeling index of much less than 5% (Figure 1M), indicating low proliferative activity. Histiocyte-like cells were positive for vimentin, CD163 and CD68 (Figures 1E-1G). CD1a and Langerin staining was negative (Figures 1H, 1L). While the S-100 protein appeared to stain a small number of cells, it was predominantly negative (Figure 1I). Staining was negative for lysozyme, glial fibrillary acidic protein (GFAP) (Figures 1K, 1N), neurofilament, CD23, CD35, CD30, factor VIII, and α-smooth muscle actin (α-SMA). Staining of CD20, CD3, CD4, and CD8 was variably positive in infiltrating lymphocytes, but negative in histiocyte-like cells. The histological and immunohistochemical features indicated a diagnosis of JXG.

**Figure 2:**
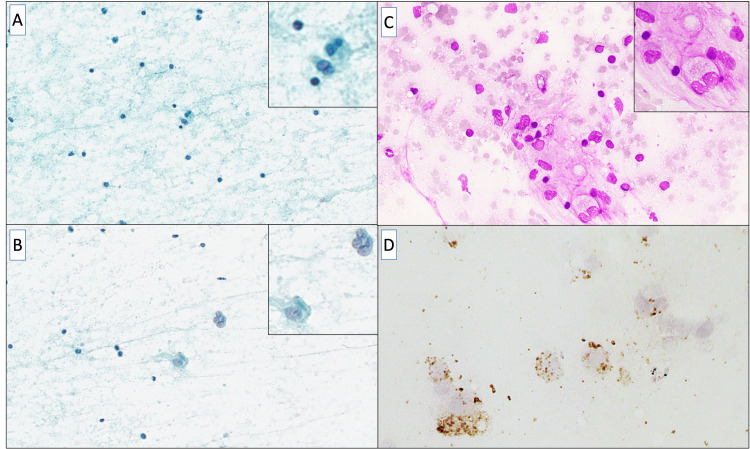
Cytologic features. (A, B) Papanicolaou-stained cytological smear presents scattered cells with a characteristic cytomorphology. The nuclei appear small, round to oval, and basophilic, while the cytoplasm seems faint and poorly defined. The inset emphasizes magnified details of isolated cells with clearer nuclear structures (Papanicolaou staining; ×100, Insets; ×400). (C) May-Grünwald-Giemsa stained microscopic image shows a heterogeneous cellular population with foamy histiocytes, scattered inflammatory cells, and occasional multinucleated giant cells. The inset provides a closer view of the cellular details, highlighting the characteristic xanthomatous cells with pale, vacuolated cytoplasm and surrounding inflammatory cells with darker-staining nuclei (May-Grünwald-Giemsa staining; ×200, Inset; ×400). (D) Immunocytochemical staining for CD68 in cytological specimens of xanthogranuloma. CD68-positive macrophages are indicated by brown staining. Immunocytochemistry shows scattered and clustered CD68-positive macrophages, including foamy histiocytes and multinucleated giant cells. The staining intensity varies among cells, with some exhibiting strong cytoplasmic positivity. The background contains a few smaller stained granules, likely representing cellular debris or fragmented macrophages (Immunohistochemical staining; ×400).

An intraoperative cytological smear with Papanicolaou staining demonstrated a low-cellularity background predominantly composed of mononuclear cells, which were scattered throughout (Figures 2A, 2B). These cells exhibited round-to-oval nuclei with smooth contours, bland chromatin, and absent or inconspicuous nucleoli. The pale, indistinct cytoplasm occasionally contained fine vacuolations, indicating histiocytic differentiation. Scattered small mature lymphocytes contributed to the mild inflammatory background, which also showed a faint hazy appearance, likely due to cytoplasmic debris or proteinaceous material. No evidence of atypia or malignancy was observed. In addition, the May-Grünwald-Giemsa stained smear of JXG showed a heterogeneous cellular population with foamy histiocytes containing pale, vacuolated cytoplasm due to lipid accumulation (Figure 2C). The background included scattered inflammatory cells, such as lymphocytes, emphasizing the diagnostic features of JXG.

Moreover, these histiocytic cells included a few foamy macrophages and multinucleated giant cells. Upon re-examination by immunocytochemistry (Figure 2D), scattered CD68-positive macrophages were observed with variable staining intensity. Some cells showed strong cytoplasmic positivity, whereas others showed a more diffuse or granular staining pattern. The background also contained small CD68-positive granules, which likely represented cellular debris or fragmented histiocytes. These cells exhibited abundant foamy cytoplasm, with densely packed CD68-positive granules. These findings supported histiocytic proliferation consistent with xanthogranulomatous inflammation.

After surgery, the patient showed significant improvement. A follow-up MRI of the cervical spine confirmed that the tumor had been completely removed, with no remaining lesions detected. At the final follow-up examination (2.5 years post-surgery), the patient had fully recovered with no tumor recurrence.

## Discussion

JXG is a rare histiocytic proliferative disorder categorized as non-LCH, which predominantly affects children. However, JXG can also appear in adults, most frequently during the third or fourth decade of life. Cases involving extracutaneous sites such as the spine are infrequent, and cervical spine involvement is extremely unusual. According to the available English-language literature, only five adult cases of cervical spinal JXG have been documented [1-5]. Among the five reported cases of cervical spine JXG in adults, the majority were located in the extramedullary region. To the best of our knowledge, our case report presents the first documented instance of cervical intramedullary spinal JXG in an adult patient. The rarity of the disease and unusual anatomical involvement posed significant challenges in diagnosis.

The diagnosis of JXG requires a histopathological examination. The most recent cytological description of JXG, published by Chauhan et al. [9], described predominantly foamy histiocytes observed throughout the papillary and reticular dermis, extending to the subcutis and interspersed with numerous Touton giant cells, lymphocytes, neutrophils, and fibrohistiocytic spindle cells. Furthermore, Grenko et al. [8] reported cytological findings of JXG from the axillary and supraclavicular lymph nodes, revealing histiocytes with reniform nuclei intermixed with lymphocytes, occasional eosinophils, and multinucleated giant cells. Most giant cells had clustered nuclei, with some displaying a wreath-like (Touton) configuration. The present case appears to correspond closely to these features. Cytological immunocytochemical evaluation plays a crucial role in reinforcing the diagnosis. Smears stained with Papanicolaou showed low cellularity, with mononuclear histiocyte-like cells exhibiting round to oval nuclei, smooth chromatin, and indistinct cytoplasm with fine vacuolations. Furthermore, immunocytochemical reexamination allowed us to focus on scattered multinucleated giant cells with CD68-positive cytoplasmic granules, further supporting the diagnosis of JXG. These findings were consistent with prior reports of JXG cytology, which emphasized the presence of histiocytes with foamy cytoplasm, Touton giant cells (although not always present), and an inflammatory background [8]. Dehner [10] observed foamy histiocytes interspersed with Touton giant cells, lymphocytes, neutrophils, and fibrohistiocytic spindle cells in mature cutaneous lesions, though these cells were less frequent in early or extracutaneous cases. Janssen and Harms [11] noted the prominence of Touton giant cells in mature lesions but their absence in early stages. Fassina et al. [12] and Bandyopadhyay et al. [13], using fine-needle aspiration cytology, confirmed the presence of foamy histiocytes and inflammatory infiltrates, though Touton giant cells were inconsistently observed. They emphasized the importance of CD68-immunopositivity in confirming the diagnosis. These findings collectively underscore the variability of Touton giant cells and highlight the critical role of immunocytochemistry in reinforcing JXG diagnosis across diverse presentations.

In JXG, histiocytes consistently express vimentin, CD68, and factor XIIIa, but lack the expression of S-100 protein and CD1a [14]. Immunohistochemistry (IHC) findings are positive for CD68 and vimentin. CD1a plays a crucial role in differentiating these tumors from LCH because LCH lesions are more aggressive and require intensive treatment. Langerhans cells are identified based on IHC positivity for CD1a. In this case, the cells were negative for both CD1a and S-100 proteins, ruling out LCH, Rosai-Dorfman disease, and indeterminate dendritic cell tumor. Moreover, the BRAFV600E mutation, which is frequently associated with Erdheim-Chester disease and is characterized by a histological appearance resembling that of JXG, was not detected. Based on these findings, a diagnosis of JXG was confirmed.

## Conclusions

In summary, we report an extremely rare case of histologically classic JXG located in the cervical cord of an adult patient. Cytology serves as a fundamental diagnostic tool for this purpose.
